# The Importance of Practicing at Home During and Following Cognitive Behavioral Therapy for Childhood Anxiety Disorders: A Conceptual Review and New Directions to Enhance Homework Using Mhealth Technology

**DOI:** 10.1007/s10567-024-00476-5

**Published:** 2024-04-14

**Authors:** Anke M. Klein, Annelieke Hagen, Lynn Mobach, Robin Zimmermann, Jeanine M. D. Baartmans, Jasmin Rahemenia, Erwin de Gier, Silvia Schneider, Thomas H. Ollendick

**Affiliations:** 1 Developmental and Educational Psychology, Leiden University, Leiden, The Netherlands; 2 Developmental Psychology, University of Amsterdam, Amsterdam, The Netherlands; 3 Pro Persona Institute for Integrated Mental Health Care, Wolfheze, The Netherlands; 4 Mental Health Research and Treatment Center (FBZ), Ruhr University, Bochum, Germany; 5 Education and Child Studies, Leiden University, Leiden, The Netherlands; 6 Trifork Amsterdam B.V, Amsterdam, The Netherlands; 7 Department of Psychology, Child Study Center, Virginia Tech, Blacksburg, USA

**Keywords:** Exposure, Homework, Child, Anxiety, Treatment

## Abstract

Practicing newly acquired skills in different contexts is considered a crucial aspect of Cognitive Behavioral Therapy (CBT) for anxiety disorders (Peris et al. J Am Acad Child Adolesc Psychiatry 56:1043–1052, 2017; Stewart et al. Prof Psychol Res Pract 47:303–311, 2016). Learning to cope with feared stimuli in different situations allows for generalization of learned skills, and experiencing non-occurrence of the feared outcome helps in developing non-catastrophic associations that may enhance treatment outcomes (Bandarian-Balooch et al. J Behav Ther Exp Psychiatry 47:138–144, 2015; Cammin-Nowak et al. J Clin Psychol 69:616–629, 2013; Kendall et al. Cogn Behav Pract 12:136–148, 2005; Tiwari et al. J Clin Child Adolesc Psychol 42:34–43, 2013). To optimize treatment outcome, homework is often integrated into CBT protocols for childhood anxiety disorders during and following treatment. Nevertheless, practicing at home can be challenging, with low motivation, lack of time, and insufficient self-guidance often listed as reasons for low adherence (Tang and Kreindler, JMIR Mental Health 4:e20, 2017). This conceptual review provides an overview of (1) how existing CBT childhood programs incorporate homework, and empirical evidence for the importance of homework practice, (2) evidence-based key elements of practice, and (3) how mHealth apps could potentially enhance practice at home, including an example of the development and application of such an app. This review therefore sets the stage for new directions in developing more effective and engaging CBT-based homework programs for childhood anxiety disorders.

Experiencing fear or anxiety is part of development and is a normal response in threatening situations. However, when fear and/or anxiety is excessive, occurs in the absence of actual danger, and interferes with daily functioning, a child is likely to meet the criteria for an anxiety disorder (American Psychiatric Association [APA], 2013). With a prevalence of 15–20%, anxiety disorders are among the most prevalent mental disorders that hinder children in their development (APA, 2013; Beesdo et al., 2009; Polanczyk et al., 2015). Children often meet criteria for more than one anxiety disorder at the same time and comorbidity with other disorders is high (Ollendick et al., 2008). Moreover, untreated childhood anxiety disorders are frequently associated with impairments in academic and social-emotional functioning and an increased risk of developing other psychopathology in adulthood (de Vries et al., 2019; Lieb et al., 2016; Rapee et al., 2009). The enormous impact of anxiety disorders on children’s development highlights the need for early and effective interventions for childhood anxiety disorders.

Over the past decades, Cognitive Behavioral Therapy (CBT) has received strong support and is considered the first-choice treatment for childhood anxiety disorders (Higa-McMillan et al., 2016; Warwick et al., 2017). CBT is inherently an integrative therapy (see Power, 2002) and therefore encompasses a broad range of treatment elements, such as exposure, cognitive restructuring, psychoeducation, parent involvement, relaxation, social skills training, mindfulness, and more, depending on the specific CBT program (e.g., Brave; Spence et al., 2006; Cool Kids; Rapee et al., 2006; Coping Cat; Kendall et al., 1990; One-Session Treatment; Öst & Ollendick, 2001). Even though CBT has been shown to be effective and the majority of children are free of their anxiety disorder(s) after treatment (Cartwright-Hatton et al., 2004), there is still room for improvement in treatment outcomes. Indeed, around 40% of youth receiving CBT are not free of their anxiety problems after treatment (James et al., 2013; Silverman et al., 2008; Warwick et al., 2017). In addition, a recent meta-analysis on relapse rates after CBT for youth anxiety showed an overall relapse rate of 10.5% across studies, and relapse rates did not differ between different primary anxiety disorder diagnoses (Levy et al., 2022). Thus, both long- and short-term treatment effectiveness could benefit from further refinement of treatment protocols.

During CBT, a significant focus is placed on extinction (or inhibitory learning), which refers to a learning process in which individuals develop a new, non-catastrophic association or representation in memory (e.g., ‘when I am confronted with a dog, nothing dangerous will happen’) that competes with the original catastrophic, fearful association (e.g., ‘when I am confronted with a dog, the dog will bite me’) (Craske et al., 2022). Considering that the old, fearful association is thought to still exist after creating the new, non-catastrophic association, there is a chance that the old association will be activated upon re-encountering the feared stimulus or situation in a new context (i.e., fear renewal; Craske et al., 2022). The chance of fear renewal can be minimized with practicing the newly learned skills in a broad range of contexts. This promotes generalization and reinforces the newly learned non-catastrophic association, thereby reducing the chance of relapse (Craske et al., 2014, 2022). The prescription and implementation of homework exercises is therefore considered crucial to learn CBT skills in different contexts and in real-life situations, outside the therapeutic context. In this manner, children learn that their feared outcome does not occur in a wide range of contexts. Indeed, many CBT programs incorporate home practice both during and after therapy in an effort to maintain treatment gains (Beck & Dozois, 2011; Öst & Ollendick, 2017; Tang & Kreindler, 2017).

One potential way to improve treatment outcome for childhood anxiety involves a renewed focus on the role of homework during and after treatment. While homework is considered an important element in most child CBT programs, few studies have specifically examined its effects on treatment outcomes, and the results are mixed (e.g., Arendt et al., 2016; Hughes & Kendall, 2007). Therefore, the overarching purpose of the current review is to provide an overview of the existing literature on homework in order to propose new directions for how homework could enhance treatment outcomes. More specifically, we focused on three aspects in our review: First, we provide a brief overview and examples of how treatment programs for childhood anxiety currently incorporate homework. Second, we describe the theoretical, evidence-based core elements that should be included in homework. Finally, we explore how digital health innovations, such as a smartphone application, could potentially facilitate the homework process. In this regard, we provide an example of the development of such an app.

## Overview of How Homework is Incorporated in Current CBT Programs for Childhood Anxiety Disorders

Most homework practice in CBT programs for childhood anxiety disorders aligns with the scope and content of areas addressed during the sessions (see e.g., Power, 2002). Given CBT’s integrative nature, homework assignments therefore reflect the broad range of treatment elements, including, among others, exposure (e.g., practicing with exposure to a feared situation), cognitive restructuring (e.g., filling out cognitive restructuring exercises), and psychoeducation (e.g., reading materials on the rationale of CBT). Most often, this means that a child attends a session, discusses, or practices a specific topic together with the therapist, and then completes the associated homework assignments. Alternatively, the homework may involve preparation for an upcoming session or continued practice with previously learned skills.

The currently used childhood CBT programs are largely based on studies and programs originally developed for adults (e.g., Beck, 1976). One of the first studies reported on using behavioral techniques to overcome childhood anxiety was reported in the 1980s (King et al., 1988) and the first formalized CBT program for childhood anxiety disorders to also include homework assignments was published in 1990 (Kendall et al., 1990). Childhood anxiety CBT programs are most often generic in nature, meaning that they are not (anxiety) disorder specific. Examples of widely used generic child CBT programs for anxiety disorders are Coping Cat (Kendall & Hedtke, 2006a, 2006b), the Friends program (Barrett et al., 2000a, 2000b, 2000c), Cool Kids (Rapee et al., 2006), Think Good – Feel Good (Stallard, 2005), Discussing + Doing = Daring (DDD; Bögels, 2008), the BRAVE program (Spence et al., 2006), and the CHILLED program (Schniering & Rapee, 2020). There are also some programs designed for specific anxiety disorders, such as the One-Session Treatment (OST) for childhood specific phobias (Öst & Ollendick, 2001; based on Öst, 1989, 1997) and the Separation Anxiety Family Therapy, also known as the TAFF program, for childhood separation anxiety (Schneider et al., 2011).

An example of one of the first generic programs where homework is integrated into the treatment protocol is Coping Cat (Kendall & Hedtke, 2006a, 2006b; Kendall et al., 1990). This program focusses on four major cognitive components: (1) recognizing anxious feelings and somatic reactions to anxiety, (2) identifying cognitions in anxiety-provoking situations, (3) developing a plan to help cope with the situation, and (4) evaluating the success of the coping strategies and self-reinforcement. Additionally, strategies such as modeling, in vivo exposure, role play, relaxation training, psychoeducation, cognitive restructuring, and reinforced practice are used. Following each weekly session, children are assigned homework tasks aligned with the content of that specific session (Hudson & Kendall, 2002). Homework is reviewed at the beginning of each succeeding session and compliance is rewarded. The full CBT program aims to teach children several skills, and the skills that are addressed during the session are also part of the weekly homework for the child. For example, the first skill that is taught in Coping Cat is affect recognition. As a homework task, children are asked to write about a situation in which they felt scared or nervous and a situation in which they felt relaxed (Kendall & Hedke, 2006a, 2006b).

Since the first CBT programs for childhood anxiety disorders were developed, several adaptations have been made. First, some programs that were only available face to face in the earlier years were later adapted to be followed at home, guided by a therapist. Often first as CD-ROM versions and later as full online programs, such as the computer assisted version of Coping Cat named Camp Cope a Lot (Khanna & Kendall, 2008), the Cool Kids program (Chatterton et al., 2019; Lyneham & Rapee, 2006; McLellan et al., 2015), the CHILLED plus program (online version of the Chilled program; Schniering et al., 2022), and the BRAVE online program (March et al., 2008, 2018; Spence et al., 2011). Several studies showed that these online programs were effective in reducing childhood anxiety (e.g., Khana & Kendall, 2010; Rapee et al., 2006; Stasiak et al., 2016; Wuthrich et al., 2012), and the BRAVE online program was also found to be as effective as the face-to-face program (Spence et al., 2011). To further elaborate on the content of one of these online CBT programs, we will have a closer look at BRAVE online. This program comprises 10 weekly child/adolescent sessions (ca. 60 min per session). The program is followed by two booster sessions, 1 and 3 months after completion of the initial program. During the 10 weekly online sessions, children and adolescents learn different anxiety management strategies together with their family from home, including recognition of the physiological symptoms of anxiety, relaxation strategies (progressive muscle relaxation, guided imagery, and deep breathing), cognitive strategies of coping self-talk and cognitive restructuring, graded exposure, problem-solving techniques, and self-reinforcement of “brave” behavior. Each session starts with a summary and quiz about the previous session and ends with a summary and quiz about the current session. Homework exercises are in line with the content of the sessions and are completed between sessions (e.g., a fear hierarchy or exposure task), and therapists provide feedback on the exercises (Spence et al., 2008).

A second adaption to the CBT programs is that some authors created modular versions of their integrated protocols. If the therapist chooses to work with a modular version of the protocol, this usually means that the child only completes the homework that belongs to the specific modules that are used. This means that not all children do the same homework assignments but may skip part of the homework or repeat some of the homework depending on their individual needs. An example of a modular program is the Discussing + Doing = Daring program (DDD, e.g., van Steensel et al., 2022). Originally, DDD was a face-to-face generic CBT program for childhood anxiety (Bögels, 2008), but was recently adapted to include separate modules (i.e., psychoeducation, task concentration, cognitive restructuring, relaxation and mindfulness, experiments, exposure, parental involvement, and relapse prevention). In the trial assessing this modular version (van Steensel et al., 2022), the modules psychoeducation and relapse prevention were mandatory in the first and last session, respectively. For all other sessions, therapists and clients collaboratively selected the modules. An advantage of working with modules is that the therapist can either skip or repeat certain sessions based on the individual needs of the child. There is some evidence suggesting that using a modular approach leads to better treatment outcomes for childhood anxiety disorders, although the evidence is still limited (e.g., van Steensel et al., 2022).

## Empirical Evidence for the Importance of Homework

In the adult literature, several studies emphasize the importance of homework and find that homework compliance predicts treatment outcome (e.g., Schmidt & Woolway-Bickel, 2000; Westra et al., 2007). Three meta-analyses underscore the significant influence of homework compliance on treatment effectiveness across various symptoms, including anxiety (Kazantzis et al., 2010; Kazantzis et al., 2016; Mausbach et al., 2010; see also LeBeau et al., 2013). For example, in a meta-analysis of 23 studies conducted between 2000 and 2008, involving 2183 participants, a small-to-medium effect size for homework compliance was found on anxiety outcomes (Mausbach et al., 2010).

In the child literature, however, the empirical evidence for the importance of homework and the effect of homework compliance on treatment outcome on childhood anxiety is scarce, with only three empirical studies, and the evidence is far less conclusive (Arendt et al., 2016; Hughes & Kendall, 2007; Lee et al., 2019). Therefore, we also included studies here on other internalizing childhood disorders (depression: Clarke et al., 1992; Shrik et al., 2013; Simons et al., 2012; obsessive compulsive disorder [OCD)]: Park et al., 2014). One of the first studies reporting on the effect of homework compliance on treatment outcome in children was on depression (Clarke et al., 1992). Clarke and colleagues (1992) did not find any evidence for the effects of homework completion on treatment outcome. A later study by Shirk and colleagues (2013) on childhood depression confirmed these results. Hudson and Kendall (2002) were among the first to discuss the importance of homework specifically for children and adolescents with anxiety disorders; however, they did not empirically test whether homework compliance influenced treatment outcome. In 2007, Hughes and Kendall were the first to empirically test the effect of homework compliance in children with an anxiety disorder with their Coping Cat program. In this study, homework compliance did not predict treatment outcome. A later study using the Coping Cat program for children and adolescents confirmed these earlier findings (Lee et al., 2019). In line with these studies, Arendt and colleagues (2016) did not find an association between homework compliance and treatment outcome for Cool Kids, another generic CBT anxiety program.

One of the first studies to provide evidence that homework compliance-predicted treatment outcome was reported by Simons and colleagues (2012). They found that homework completion between treatment sessions predicted treatment outcome in adolescents with depression. Another study by Park and colleagues (2014) found evidence for the importance of homework compliance when studying the effects of d-cycloserine (DSC; DSC is used as an adjunctive medication and has been demonstrated to facilitate fear extinction) on homework compliance in a group of children with obsessive–compulsive disorder. They did not find that children who took DSC had a higher homework compliance than children who did not take DSC. However, they did find a significant main effect of homework compliance across conditions. Children with higher homework compliance showed more improvement in OCD symptom severity.

## Reasons for these Mixed Findings

The previous section showed that, while the addition of homework practice in adults consistently adds to the effects of CBT, for children, studies are scarce, and the findings are mixed at best. Although these findings seem to downplay the importance of homework for CBT in childhood anxiety disorders, such a conclusion would be premature, as the evidence base is too small to draw strong conclusions. There are many important lessons that can be learned from the studies conducted so far and from the larger evidence base in adults. This section will critically review several methodological and theoretical factors addressing the mixed results on the effect of homework. In the following section, recommendations for homework programs are formulated based on these factors.

First, the mixed findings could be related to child, parent, and therapist factors. In general, individuals do not spend a lot of time on their homework assignments due to a variety of reasons, including lack of time, low motivation (Carroll et al., 2005; Detweiler & Whisman, 1999; Nock & Kazdin, 2005), lack of interest (Merry et al., 2012), not fully understanding the homework (Dozois, 2010), or (emotional) avoidance (Dobson et al., 2014; Hofmann, 2013). Additionally, especially in children, it is suggested that parents may also play a role in homework adherence (Arendt et al., 2016; Lee et al., 2019). Presumably, parents play an important role in facilitating homework exercises by providing support, resources, or simply enabling access to certain situations for exposure to occur (Clarke et al., 2015). A lack of support or resources may be one of the reasons why homework programs are not as effective as they could be (see also, Wilansky et al., 2016). Another reason that homework might not be as effective as it could be is due to therapist factors. For example, therapists might feel that they do not have sufficient time during the therapy session, perceive resistance of the child to do homework, or be concerned that the child is not able to handle the homework (Kelly & Deane, 2011). It seems important to take these child, parent, and therapist aspects into account when developing homework programs and evaluating the effects of homework in future studies.

Second, only a handful of studies investigated the effect of homework on treatment outcome in childhood internalizing problems, including anxiety, and the large majority of these studies mention methodological issues as one of the reasons for the mixed findings. One of the issues that was discussed includes the use of different dependent variables, such as the time spent on homework, proportion of task completion, and number of tasks completed. Additionally, there was large variability across studies in when the effect of homework was measured, for example, during treatment, post-treatment, or during follow-up. In addition, another study suggested that it might be more difficult to reliably measure the amount of time spent on homework in children, as children might find it challenging to keep track of their practice time (Arendt et al., 2016). These different outcomes, differences in time of measurement, and reliability of the measurements may have led to different and therefore inconsistent findings (see also, Kazantzis et al., 2016). Alternatively, focusing on qualitative ways of looking at homework might yield different insights into possible strategies and outcomes. For example, a child might not spend much time on homework, but the time spent might be very meaningful and high in quality (see also, Kazantzis et al., 2016). Indeed, a study on adults with a panic disorder (Schmidt & Woolway-Bickel, 2000) found that the quality of homework was a better predictor of treatment outcome than the quantity of the homework. This hypothesis, however, has not been examined in children.

Third, theoretical factors should be discussed as possible explanations. As discussed above, the content of CBT-based homework programs generally reflects the broad scope of elements that forms the generic CBT programs for childhood anxiety. This is based on the principle that session content should be practiced at home to ensure adequate mastery of skills and to stimulate generalization of skills to the home environment and beyond (Kazantzis & L’Abate, 2005). However, due to a general lack of mechanistic research focusing on working mechanisms of CBT for child anxiety, it is unclear to what extent all these individual elements add to the efficacy of CBT for anxiety (Kazdin, 2018). It could theoretically be expected that certain homework assignments may tap more into core working mechanisms of CBT, whereas other homework assignments may not.

Leading theories of both the maintenance of anxiety disorders and the working mechanisms of CBT find common ground: a focus on correcting dysfunctional cognitions and anxious expectations using corrective techniques is key for treatment success (e.g., Beck, 1976; Craske et al., 2022; Spence & Rapee, 2016). In this regard, exposure is considered the most effective working element of CBT for childhood anxiety and involves the repeated confrontation with feared stimuli or situations in the absence of the feared outcome to correct these dysfunctional, anxious expectations, and beliefs (Craske et al., 2022; Crawley et al., 2013). This conclusion has found support in several studies in adult anxiety disorders (e.g., Craske et al., 2022; Pittig et al., 2023) and a handful of studies in the childhood anxiety disorders field (Kendall & Treadwell, 2007; Mobach et al., 2021; Whiteside et al., 2019). Practicing with feared stimuli or situations allows for generalization and successful experiences in developing non-catastrophic associations that may maximize treatment outcomes (Bandarian-Balooch et al., 2015; Cammin-Nowak et al., 2013; Kendall et al., 2005; Tiwari et al., 2013).

There is no theoretical reason to assume that homework assignments have a fundamentally different working mechanisms than CBT programs themselves. Therefore, if some aspects of a homework program are more important than others (e.g., exposure), the large variability (reflecting the full range of CBT elements) in homework programs included in the studies reviewed above may dilute the effects of certain homework assignments that reflect these supposed working elements of CBT. Indeed, some studies suggested this hypothesis as a possible explanation for their insignificant findings (Arendt et al., 2016; Hughes & Kendall, 2007; Parker et al., 2023). This reasoning was partly confirmed in one of the few studies that did find an effect of homework on treatment outcome (Park et al., 2014). In this study, the tested homework program almost exclusively consisted of exposure-related exercises and relapse prevention. Also, other studies in adults found evidence for the fact that homework adherence was found to be more important at certain times during treatment, such as at the start of treatment (Westra et al., 2007), which might indicate that some aspects or timing of homework might be important (Simpson et al., 2011; Westra et al., 2007).

Keeping in mind that (1) from a theoretical perspective, adherence to homework exercises during and after treatment are crucial and (2) the difficulties observed in clinical practice in obtaining sufficient homework compliance (see e.g., Hudson & Kendall, 2002; Lundkvist-Houndoumadi et al., 2016), a logical conclusion and recommendation would hence be to focus homework exercises on the theoretically important and empirically supported working elements. That is, exposure to break the avoidance cycle and correct dysfunctional cognitions and anxious expectations.

## Key Elements of Homework for Childhood Anxiety Disorders

In the previous section, three overarching themes were discussed in relation to a general lack of homework effects for childhood anxiety in the studies conducted so far: child, parent, and therapist factors; methodological factors (e.g., quality and quantity of homework); and theoretical (homework content) factors. In this section, we have translated the shortcomings addressed above into structural, key elements that should be addressed to improve CBT-based homework programs for child anxiety. To this end, we have compiled a list of 11 key elements and conditions that evidence-based homework programs should consider and/or incorporate based on the literature. We have grouped these elements according to the three overarching themes discussed above, starting with the theoretical factors and working toward more specific child, parent, and therapist factors. See Table 1 for a concise overview of the key elements described below.

**Table 1:** Overview of recommended key elements for homework programs in childhood anxiety treatment

Element	Description of the element
*Theoretical factors*	
1. Setting the stage for expectancy violation	Formulate exercises focusing on expectancy violation and inhibitory learning rather than fear reduction. Maximize prediction error, remove distractions and safety behaviors, and include mental rehearsal
2. Multiple contexts	Conduct exercises in various internal and external contexts to generalize learning and prevent fear renewal
3. Flooding vs. gradual exposure	Choose between gradual or flooding exposure techniques, considering child-specific needs
4. Mental rehearsal	Include mental rehearsal between sessions to strengthen learning, retrieval and enhance motivation for exposure exercises
*Methodological factors*	
5. Personalized exposure exercises	Tailor exposure exercises to match the child's skill level and individual characteristics, considering the heterogeneity of anxiety disorders
6. Psychoeducation	Provide psychoeducation to provide continuous explanation on the why and how of exposure to increase motivation and to ensure adequate and continuous practice at home
7. Contingency management	Incorporate rewards and reinforcements to stimulate engagement in homework exercises
*Child, Parent, and Therapist factors*	
8. Therapist guidance and clear instructions	Provide therapist support in various forms to enhance program usage, treatment outcomes and motivation
9. Self-monitoring	Include self-monitoring in homework programs to guide reflection on learning outcomes and enhance expectancy violation and memory consolidation
10. Involvement of parents	Involve parents in facilitating homework completion by providing instructions, materials, and psychoeducation, emphasizing their role in exposure exercises
11. Co-creation and user-friendliness	Design user-friendly homework programs through co-creation with end-users, considering diverse backgrounds and developmental stages for increased effectiveness

### Theoretical factors:

1. ***Setting the stage for expectancy violation*.** In formulating the homework exercises, a focus on expectancy violation and corrective learning instead of on fear reduction is key. Craske and colleagues (2022) recently formulated a theoretically and empirically supported exposure plan with key points that should be addressed when planning both guided and unguided exposure. The following principles should be adhered to maximize prediction error, remove distraction(s), remove safety behaviors and signals, and include mental rehearsal after the homework exposure exercises (see key element 9). A well-designed exposure exercise should include a focus on testing a specific anxious expectation about a feared stimulus or situation that the child rates as very believable (i.e., has a high threat expectancy rating). The exposure exercise should be set up in such a way that it includes a high chance of experiencing a disconfirmation of the anxious expectation. Maximizing the prediction error between what the child expects will happen (e.g., ‘The dog will bite me’) and what happens in reality (e.g., the dog licks the child’s hand and does not bite) is very important in increasing these chances. To reach this goal, it is paramount that safety signals and safety behaviors are removed (as much as possible) and no cognitive interventions aimed at reducing the believability of the threat expectancy (e.g., ‘how many children get bitten by a dog every year?’) precede the exposure exercise. It is beyond the scope of this article to review all principles in detail; please see Craske and colleagues (2022) for an in-depth discussion. Adequate therapist guidance and therapist modeling during guided exposure exercises within sessions can help with adhering to these rules (see key element #4). In addition, training the parents to guide the child with the more difficult homework exercises or ensuring that the necessary materials or situations are facilitated is necessary for a successful exposure exercise (see key element #11).
2. ***Multiple contexts*.** In close connection to key element #1, it is important that exposure exercises are conducted in a variety of internal and external contexts (e.g., at home, different varieties of the same stimulus (e.g., different types of dogs/spiders) outside, different public places, different emotional states), and in a variety of circumstances (e.g., with/without parents, siblings present) to generalize and consolidate the learning coming out of the expectancy violation: what the child expects will happen does not take place in many different contexts and circumstances. Studies have shown that varied practice decreases the chance of fear/context renewal and, in the end, relapse (e.g., de Jong et al., 2019; Jacoby & Abramowitz, 2016; Maren et al., 2013). Homework exercises play an important role in achieving this goal, as the therapeutic context is often limited to practicing only a couple of these contexts and circumstances.
3. ***Flooding versus gradual exposure*. **While guidelines for adult exposure exercises have incorporated ‘flooding’ instead of gradual exposure for a longer time, child CBT programs still mostly include gradual exposure using a hierarchy of exposure exercises, starting with the easiest and least anxiety-provoking exercise and ending with the most difficult and fear-inducing exercise (Davis et al., 2019; Mobach et al., 2020). For spider phobia, an example of gradual exposure is starting with looking at pictures of spiders before progressing to more fear-provoking exercises, such as looking at live spiders or handling them. Flooding, in contrast, does not follow a fear hierarchy. Instead, it may incorporate starting with exercises that provoke more fear, potentially including the intended end goal of therapy, such as handling live spiders. Flooding has been associated with larger reductions in dysfunctional anxious expectations during exposure exercises in adults with anxiety disorders (Craske et al., 2022). However, in child anxiety, to the best of our knowledge, there have not been any studies specifically testing flooding versus gradual exposure in relation to treatment outcome or reduction in critical maintenance mechanisms of anxiety disorders. This being said, a study by de Jong and colleagues (2023) has compared gradual exposure in small steps versus exposure in larger steps. The results indicated that large steps were not more effective than taking small steps in reaching the child’s goals and in anxiety severity at post-treatment. The authors discuss the likely explanation that taking smaller steps resulted in children being exposed to a larger variety of fearful stimuli/situations, which (in relation to key element #1 and #2) likely led to more generalization of the learned outcomes. In conclusion, these findings lead to repetition of the message above that abiding by the expectancy violation set-up in combination with exposure in multiple contexts is important. Nonetheless, more research on the possible differences in efficacy between flooding and gradual exposure in childhood anxiety should be included on the agenda for future research.
4. ***Mental rehearsal*.** Including mental rehearsal in-between sessions and before and after homework exercises can act as an augmentation strategy to exposure-based exercises. Mental rehearsal has two important advantages: it stimulates and strengthens retrievability of the learning experience (compared to the original fear association) from memory and enhances motivation for future exposure exercises (McGlade & Craske, 2021). Homework programs can incorporate various forms of mental rehearsal and memory consolidation, including taking pictures/videos of successful exposure experiences and including the initial anxious expectation and the newly formulated realistic expectation. These tools can stimulate the child to reflect on these experiences and enhance consolidation into memory, which may increase feelings of self-efficacy and can aid in restructuring dysfunctional cognitions (Beidas et al., 2010). For possibilities in including mental rehearsal in exposure practice and a discussion on the potential working mechanisms of mental rehearsal (e.g., prolonged [imaginal] exposure), see also McGlade and Craske (2021).

### Methodological factors

5. ***Personalized exposure exercises*.** Guidelines for formulating homework exercises explicitly include personalizing the exposure exercises to ensure that the exercises match the skill-level and personal characteristics of the child (see Tompkins, 2002, for guidelines). Personalizing exposure exercises acknowledges the heterogeneity of each anxiety disorder and each child (Peterman et al., 2015; Wang et al., 2017) and the large variety of anxious expectations children with anxiety disorders present with during treatment. For example, not all children with a social anxiety disorder fear negative evaluation by adults (APA, 2013). Giving all children with a social anxiety disorder an exercise focused on exposure to possible scrutiny by an adult would fail to meet the needs of the child that does not feel fearful for this exposure assignment, possibly resulting in a lack of commitment to the homework exercise. Integration of an adequate case conceptualization is therefore paramount.
6. ***Psychoeducation*.** Psychoeducation plays a large role in the beginning of exposure-based CBT and is hence included in all CBT protocols. Psychoeducation is crucial to increase understanding of how anxiety complaints are maintained and how exercises can help the child to reduce their anxiety complaints. It is a way of preparing the client for therapy by providing information on the complaints and treatment rationale. Additionally, it can help with normalization, establishing a good working relationship between therapist and client and increasing engagement with therapy (see Grills et al., 2023 for an extensive review on the role of psychoeducation in childhood CBT). Starting treatment with psychoeducation can thus increase motivation in both children and parents and ensure client and therapist are in agreement on the treatment goals and approach. However, the importance of continuous psychoeducation on the how and why of exposure exercises to ensure adequate practice at home is often underestimated. For example, repeating the message that safety behaviors play an important role in maintaining anxiety just before an exposure exercise could motivate the child to leave their safety behavior behind when doing the exposure exercise. Additionally, providing easy access to psychoeducation about homework- and CBT-related topics in a homework program offers low-level support to parents and their child whenever they need to have access to information when outside of the therapeutic context. It is important to mention that there is evidence for the effectiveness of psychoeducation as an intervention by itself (e.g., Baourda et al., 2022), but the added value of psychoeducation in relation to homework has not yet been tested.
7. ***Contingency management*.** Contingency management is a standard component in treatment for childhood anxiety (e.g., Kendall, 1990; Podell et al., 2010; Rapee et al., 2009) and should be considered an important component of homework programs as well. Stimulating a child’s engagement in homework exercises, however, should be based on theory (Kazantzis & Miller, 2022). Operant conditioning principles have guided engagement principles in therapy for the last several decades (e.g., Becker et al., 2012; Skinner, 1968), stating that behavior that is reinforced is more likely to be repeated in future. Incorporating rewards and reinforcements can be done in various ways, including positive feedback after completed exercises and allowing the child to choose rewards (e.g., selecting what to eat for dinner or engaging in family activities). Contingency management is also often easily implemented in mHealth programs, where gamification is gaining momentum (e.g., Dennis & O’Toole, 2014). Gamification of mHealth for homework programs has the potential to include gaming elements (e.g., unlocking fun games after successful completion of homework exercises or collecting points) that have been associated, among other things, with improved adherence to health interventions and enhanced intrinsic and extrinsic user motivation (Dennis & O’Toole, 2014; Ferreira et al., 2014). Directly connecting a child’s goals to a certain, self-chosen reward can, therefore, stimulate the child to reach their goals.

### Child, parent, and therapist factors

8. ***Therapist guidance and clear instructions*.** Homework programs can be self-guided or therapist guided, which may also vary depending on the phase of the therapy that the homework program is used in (during treatment versus after treatment). Therapist-guided homework programs can include all sorts of different forms of support, ranging from automated reminders of the completed homework exercises to regularly scheduled telephone contact with the therapist. To the best of our knowledge, no research has been done specifically on the role of therapist support in CBT homework programs. In general, studies have shown that regular contact with a therapist during online or bibliotherapy CBT formats substantially increases usage of the program, improves treatment outcomes, and is related to lower dropout rates (see for a review, Baumeister et al., 2014; Marks & Cavanagh, 2009; Stasiak et al., 2016). Specifically, for mHealth-supported treatment, inclusion of therapist support tends to be more effective as it increases accountability and motivation (Wright et al., 2019).
9. ***Self-monitoring*. **Self-monitoring can take on many forms, including asking the child for specifics on the exercise (e.g., ‘what did you do during the exercise?’) and how the child feels (e.g., ‘how anxious are you now?’). Although many of these questions are helpful, key questions that should be asked focus on what the child has learned with regards to their anxious expectation to enhance expectancy violation (Craske et al., 2022, see also key element #1) and memory consolidation (Beidas et al., 2010, see also key element #4). Including some form of self-monitoring in a homework program guides the child toward what needs to be consolidated in learning before and after the exercise, can greatly enhance reflection on what is learned (i.e., ‘did your anxious expectation come true’), and holds an important place in a self-guided exposure exercise. In this way, self-monitoring is a means to an end, namely enhancing expectancy violation and memory consolidation.
10. ***Involvement of parents*. **Although the inclusion of parental involvement has not led to better treatment outcomes on the treatment level (see for a systematic review, Byrne et al., 2023) for childhood anxiety, involving parents could enhance the likelihood of completing homework exercises. Parents play an important role in facilitating the child with the right materials and situations (e.g., arranging a car ride to a certain setting or ensuring that siblings are out of the house to decrease distractions) outside of the treatment context. Involving parents early in the treatment process as ‘facilitators’ to ‘transfer control’ (Ginsburg et al., 1995; Wei & Kendall, 2014), giving parents instructions on what to look out for during the exposure exercises (e.g., safety behaviors), and directing them to the homework forms to be completed before and after the exercise can be effective (Manassis et al., 2014) and facilitate (qualitatively good) homework completion. It is crucial, however, to remind parents of the importance of their role in the exposure exercises, provide psychoeducation on family accommodation, and highlight the possibility of acting as a safety person to ensure setting the stage for adequate expectancy violation (Byrne et al., 2023; Craske et al., 2022; Lebowitz et al., 2016).
11. ***Co-creation and user-friendliness*.** Regardless of the format of the homework program (e.g., paper based, online, or smartphone application based), the homework program should be user-friendly and based on generic guidelines for improving homework outcomes (Dozois, 2010; Wilansky et al., 2016). These guidelines include, among others, the importance of shared decision-making, formulation of clear and specific exercises, and the discussion of dysfunctional attitudes related to non-compliance (e.g., ‘I can’t do homework, because….’). Many of these guidelines follow the general process of co-creation. Co-creation plays an important role in the formulation of homework assignments, as well as in formulating the homework program itself. Treatment-seeking children and their families represent a wide range of different backgrounds, developmental stages, and wishes. Co-creating homework programs with these end-users (i.e., the child and their parents, therapists) can increase the chance that the homework program format has an easy interactive format and can be easily incorporated into a child’s life and treatment program. A user-centered iterative development process that involves all end-users, including children, families, researchers, and therapists (and IT specialists in the case of mHealth), is therefore important and increases the chance that children and their families adequately benefit from their exposure exercises (McCurdie et al., 2012).

## Toward a New Way of Incorporating and Delivering Homework in Current CBT Programs

When developing and evaluating new ways to incorporate homework in current CBT programs for childhood anxiety disorders, it is important to consider the different aspects described in the previous sections. The key question is how to optimally integrate these 11 elements into existing CBT programs and how to best deliver homework. Historically, homework programs have been delivered through paper-and-pencil methods, often in the form of booklets or loose forms provided to a child after a session. One option is to directly adapt these paper-and-pencil booklets. An advantage of adapting the booklets is that it might be less work, the child can easily go back in the booklet or look at it after treatment, and the child can easily show the booklet to others. Disadvantages of these programs, however, are that the therapist have limited insight into the child’s activities at home and cannot easily help the children at home when the child is facing problems. Additionally, the child needs to remember to bring the paper-and-pencil booklets, and the booklets are usually printed and follow a standard order, which might not suit every child.

Another option is to leverage technological solutions for the delivery and adaptation of existing homework programs. While online CBT programs already incorporate technology and can utilize the aforementioned 11 points to further enhance their programs, face-to-face programs could also benefit from incorporating technological solutions as an add-on to their existing programs, facilitating home practice. Blended care, such as mHealth apps, holds the potential to overcome some of the challenges outlined in the previous section (Tang & Kreindler, 2017). For instance, an app could provide guidance on exercises and necessary preparations, better individualize the program by selecting or creating exercises tailored to the specific child, provide information about home practice, send reminders, incorporate motivational game elements, and track progress to provide therapists with insight into the homework process (Berry & Lai, 2014; Muroff & Robinson, 2022). Additionally, apps have certain advantages over online environments; they are generally more user-friendly, can be used offline (unlike online environments that always require an internet connection), and can store photos and videos only on the child’s private device, avoiding the need for uploads to an online environment, which is a large advantage with regard to privacy.

The use of mHealth apps is indeed on the rise, reflecting the growing interest in leveraging technology to enhance mental health care (Hollis et al., 2017). Currently, a multitude of Mhealth apps are available, offering exercises to address anxiety complaints. However, these apps are seldomly tested for effectiveness. Despite the consensus about the need for an evidence-based approach to childhood anxiety (Higa-McMillan et al., 2016), a review about childhood anxiety apps by Bry and colleagues (2018) concluded that approximately half of the publicly available apps featured only one of the evidence-based features listed above and merely about one-fourth included two or more evidence-based features. Unfortunately, none of these apps cover the complete range of evidence-based features. Since the publication of Bry’s et al.’s review in 2018, there have been no notable improvements in this area.

Currently, several mHealth apps are available for childhood anxiety that were developed within the academic setting, including MindClimb (Newton et al., 2020), REACH (Stoll et al., 2017), SmartCAT 2.0 (Silk et al., 2020), and Anxiety Coach (Carper, 2017; Whiteside, 2016; Whiteside et al., 2019). These apps were designed as add-ons to individual or group-based CBT, primarily aiming to assist youth in completing homework assignments between therapy sessions. Each of these apps incorporates some of the eleven mentioned aspects discussed earlier. For instance, all three apps feature a form of self-monitoring; MindClimb uses questions before and after each exercise and provides an overview of schedules events (Newton et al., 2020), REACH uses daily diary reporting (Patwardhan et al., 2015; Stoll et al., 2017), and Anxiety Coach tracks anxiety levels (Carper, 2017; Whiteside, 2016; Whiteside et al., 2019). They also include contingency management, such as positive messages and points are awarded for an event ladder completion in MindClimb (Newton et al., 2020), and digital points in SmartCAT 2.0 (Pramana et al., 2018; Silk et al., 2020). However, there are still important evidence-based elements that are either missing or under development. Only Anxiety Coach has consistently prioritized exposure as its main focus, utilizing a database of non-individualized exercises. The app does not allow for fully tailored fear hierarchies, but it does enable therapists to include individualized exercises (Carper, 2017; Whiteside, 2016; Whiteside et al., 2019). It should be noted, however, that SmartCAT 2.0 has made significant advancements in this regard. In contrast to the initial version, it now includes individualized exercises suggested by the therapist (Pramana et al., 2018; Silk et al., 2020). Since these apps are typically used as an add-on to therapy with a professional, it is crucial to comprehensively evaluate the full programs. This is particularly important as the quality of elements, such as exposure exercises, can vary significantly, and the mere inclusion of exposure exercises does not guarantee alignment with the key elements described earlier.

Thus, while current traditional CBT programs address some important aspects of homework and recently developed apps incorporate certain features, it is noteworthy that no single program currently integrates all essential elements while emphasizing the key working elements of CBT. Moreover, it is important to acknowledge that the effectiveness of these programs depends on how well therapists are trained in their ability to connect the face-to-face sessions to the homework program. What is needed is a comprehensive treatment package that integrates face-to-face or online treatment with an evidence-based homework program, possibly delivered through an app. This approach would ensure that all therapists undergo thorough training to maximize its effectiveness. The next section aims to bridge this gap by presenting a cohesive and well-integrated program, covering the in-session program, homework program, and how therapists are trained to seamlessly integrate these components into one comprehensive package.

## Development of a New Way to Enhance Homework in CBT for Childhood Anxiety Disorders

In order to address the 11 key features described before, a new homework program, The Kids Beat Anxiety (KibA) homework program (Klein et al., 2023), was recently developed as an add-on to an existing childhood CBT program. Specifically designed to enhance the One-Session Treatment (OST) protocol for childhood-specific phobias (Ollendick et al., 2015; Öst & Ollendick, 2001), this comprehensive OST program starts with an extensive intake to assess the specific phobia and identify any additional concerns. Next, a cognitive behavioral analysis is conducted to fully understand the child's dysfunctional beliefs and factors influencing their fear, such as context, companions, or the size of the feared object. The cognitive analysis is followed by a three-hour massed exposure session, a key part of OST. During this session, children engage in practice with various stimuli (e.g., three different dogs of different sizes and activity levels) under the therapist’s guidance, with the aim of achieving significant progress (for more details, see Öst & Ollendick, 2001; Ollendick et al., 2015). To sustain and extend these improvements, continued practice outside of therapy is vital, and this is where the KibA homework program comes into play.

One week following the massed exposure session, the therapist and the family reflect together on the progress made in the session and the therapist emphasizes the importance of continuing practice at home for at least four weeks. During this session, the therapist explains the homework procedure and provides guidance on what to do in case of a setback. The KibA app, primarily focusing on exposure, becomes an essential tool during this period.

During the session, the therapist and the child (along with parents) collaboratively create 10 individualized exposure exercises that focus on expectancy violation and corrective learning (key elements 1, 5, 10). Each exercise includes three difficulty levels to create multiple contexts (key element 2). For example, an exercise might involve “walking to the playground next to the dog park,” with levels ranging from walking with a parent during peak dog-walking times (bronze) to going alone during a quiet period (silver) or during busy dog-walking hours (gold). The child is allowed to freely choose from the first three exercises and whether they want to do the easier or more difficult version of an exercise. After completing 5 out of 9 levels of the first three exercises, the next three exercises are unlocked. In this way, gradual exposure is combined with flooding (key element 3). Prior to and following each exercise, children answer different questions to self-monitor their result (key element 9) and to be able to challenge their expectancies (key element 1). Besides discussing the individualized exposure exercises, the therapists and the family also discuss individualized rewards that can be earned by completing the exposure exercises and its associated goals. An example of a goal is to take photos and videos during an exposure exercise and to later re-watch these photos and videos which help with mental rehearsal (key element 4). The child earns coins by completing exposure exercises and achieving goals, which they can exchange in the store for a reward. Parents are asked to provide a surprise reward, which the child can earn by completing the program (key element 7). In addition, the app includes instructions and psychoeducation in case the child forgets anything about the instruction or has a setback (key element 6).

Children use the app for four weeks at home with the goal of practicing on a daily basis, and the therapist provides weekly contact to discuss progress and to address potential challenges (key element 8). Importantly, all therapists receive training in the OST procedure and app usage, including guidance for massed exposure sessions and examples of suitable exercises and rewards. Initial case supervision and ongoing sessions are offered to ensure effective implementation. The app was built in co-creation and is currently tested on effectiveness in a randomized controlled trial (key element 11).

**Fig. 3:**
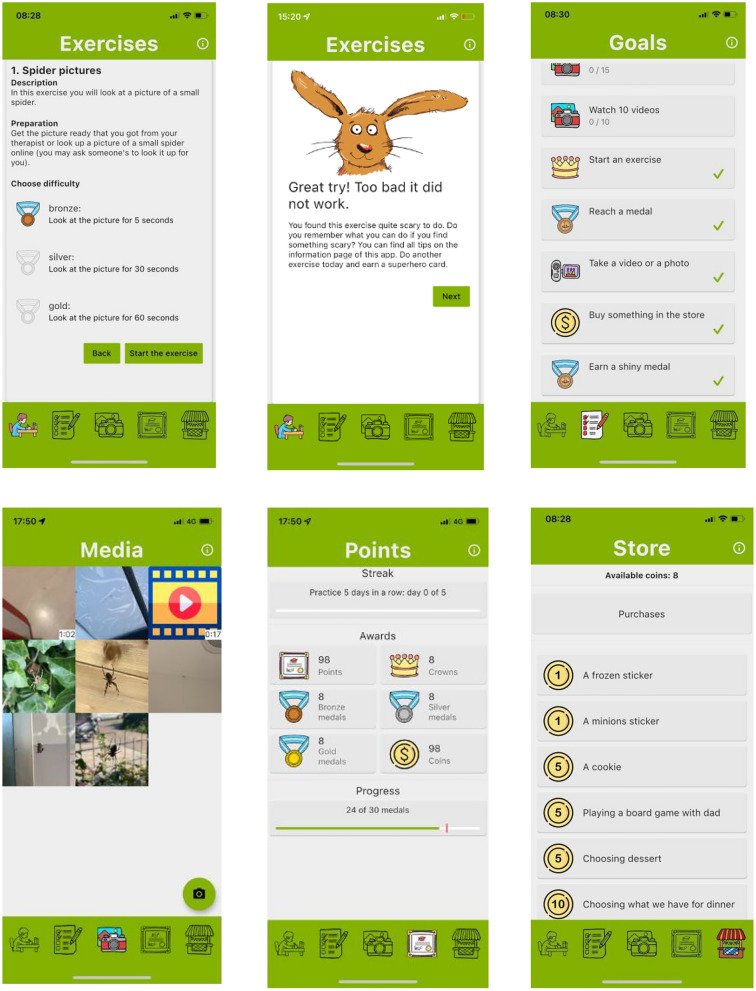
Final version of the KibA homework app

Please find a further elaboration of all features of the KibA homework program based on the 11 recommendations and its development process in appendices A and B. Appendix A provides a comprehensive understanding of how this homework program is designed, and appendix B provides an overview of the development and implementation. A summary of the key elements is also provided in Tables 2 and 3, and Fig. 3 provides screenshots of the app, highlighting its main features.

**Table 2:** Components of the KibA homework program

Component	Description of the components
Therapist training	Therapists who participate in the current RCT follow two workshops on delivering one-session treatment in combination with the KibA-app. They study the treatment and app protocol along with additional materials that include examples. All therapist treat at least one case under the close supervision of TO and/or AH. Regular supervision sessions are scheduled and available on an as-needed basis for subsequent cases
OST including cognitive analysis	All children will receive a 3-h massed exposure session, following an extensive cognitive analysis and regular intake following the OST protocol from Öst and Ollendick (2001)
KibA-app	Children practice for four weeks at home with the assistance of the KibA-app. During this period, they have a weekly phone call with their therapist to address possible challenges. The app is described in more detail in Table 3 below
Evaluation	Progress is evaluated after the home-practice period. If needed booster sessions, prolonged home practice, or alternative care can be offered

**Table 3:** In app features of the KibA homework program

Features	Corresponding key elements	Description of the feature
Individualized exposure exercises	Expectancy violation, multiple contexts, flooding vs gradual exposure, personalized exposure exercises, co-creation	Therapists and children together create ten individualized exposure exercises with three difficulty levels (easy, medium, hard). Exercises are created based on the cognitive behavioral analysis of the child’s dysfunctional thoughts, the child’s avoidance behavior, and the progress that has been made during the in-session exposure
Questions prior and following each exercise	Self-monitoring, Expectancy violation	Children answer questions prior and following each exposure exercise (e.g., “How do you feel right now,” “I think something awful or dangerous will happen during the exercise,” “Did something awful or dangerous happen?”) to challenge and disconfirm dysfunctional beliefs, and monitor feelings
Media library	Mental rehearsal	Children take pictures/videos of successful experiences and watch these pictures/videos in the media library
Goals + Coins + Store	Contingency management	Children’s successful exposure practice (e.g., petting a dog) and reaching goals (e.g., “Collect 50 points,” “Watch three videos,” “Collect a medal,” “Streak of five days practice,” “Buy something from the store”) is reinforced by the earning of digital coins, medals, and crowns. Coins act as tokens to pay for individualized reinforcers in the store (e.g., choosing dessert, 15 min more screen time, doing something fun in the weekend). Children can earn a secret surprise reward by completing at least 25 out of 30 exercises
Information tab	Psychoeducation, guidance, and user-friendliness	Children can read texts and watch videos with psychoeducation about what to do in case of a setback or high levels of anxiety. The information tab also contains links to support pages with (video) instructions and a FAQ section
Reminders	Guidance	Children receive a practice reminder message once each day
Online dashboard	Therapist guidance	Therapists access the online dashboard to add/adjust individualized exposure exercises and individualized reinforcers, monitor children’s progress, and app usage
Design	Co-creation, user-friendliness	Scientists, therapists, children, parents, and IT specialists were involved in a user-centered iterative development process with feedback sessions to ensure a user-friendly app with special attention to colors and graphics that are attractive to children

## Discussion

The purpose of the current review was to give an overview of the existing literature on CBT-based homework programs for childhood anxiety in order to provide new directions for enhancing treatment outcomes utilizing homework programs. More specifically, we focused on three aspects in our review. First, we provided a brief overview and examples of how treatment programs for childhood anxiety incorporate homework. Following this, we presented an overview of the empirical literature available on the effect of homework on treatment outcomes in childhood CBT programs. Second, we described the key elements that are important to be included in homework based on the available evidence. Third, we explored how digital health innovations, such as an app, could facilitate the homework process, including an example of the development of such an app.

The literature review indicates that CBT programs, including programs for childhood anxiety disorders (e.g., Bögels, 2008; Kendal & Hedtke, 2006a, 2006b; Ollendick et al., 2015; Rapee et al., 2006; Spence et al., 2006), have generally emphasized between-session and post-treatment homework practice. However, most programs for childhood anxiety disorders are generic in nature, and homework often directly mirrors the broad range of topics that are addressed during the sessions. While there are enough reasons to incorporate homework in childhood CBT programs and adult studies found small-to-medium effect sizes for homework compliance on anxiety outcomes, empirical research in childhood anxiety disorders is scarce (we only found three studies that specifically focused on childhood anxiety; Arendt et al., 2016; Hughes & Kendall, 2007; Lee et al., 2019). We therefore focused on empirical research in childhood internalizing disorders in our review, revealing mixed results. Several reasons were mentioned that could explain these mixed results including the (1) (theoretical) content that is addressed during the homework, (2) methodological issues, and (3) child, parent, and therapist factors. Clearly, more research is needed to investigate the effects of homework on treatment outcomes in childhood anxiety disorders. These considerations can guide the optimization of homework program content and inform future studies aimed at building an evidence base for CBT-based homework programs for childhood anxiety.

A first step in optimizing homework programs is to critically evaluate their content, considering both theoretical foundations and empirical evidence regarding potential active components of homework. Empirical studies suggest that certain aspects of homework may be more effective than others (Park et al., 2014; Simpson et al., 2011; Westra & Dozois, 2007). For instance, homework activities like reading through psychoeducation material or completing cognitive restructuring exercises may be less effective than engaging in actual exposure exercises, which could more optimally make use of the efforts invested by children and their families in completing homework exercises. This aligns with recent developments in both adult and childhood CBT, emphasizing a growing focus on the active elements of CBT. The consensus in adult CBT studies for anxiety is that cognitive restructuring and exposure are primary, active elements driving CBT, as demonstrated in various studies (e.g., Beck, 1976; Craske et al., 2022). Unfortunately, there is currently a lack of studies in children investigating the working mechanisms or crucial elements of CBT for childhood anxiety (e.g., Kendall & Treadwell, 2007; Mobach et al., 2021; Radtke et al., 2021; van Steensel et al., 2022). Clearly, more research is needed to better understand the factors influencing treatment outcomes, especially as most homework follows the in-session content. Particularly considering that (1) from a theoretical perspective, adherence to homework exercises during and after treatment are crucial and (2) the difficulties observed in clinical practice in obtaining sufficient homework compliance (see, Hudson & Kendall, 2002; Lundkvist-Houndoumadi et al., 2016). A logical conclusion and recommendation would be to design homework exercises based on the theoretically important and empirically supported working elements. That is, exposure to correct dysfunctional cognitions and anxious expectations.

Secondly, in addition to critically assessing the content of current CBT programs and associated homework, we must consider child, parent, and therapist factors. In general, practicing at home can be challenging due to factors, such as low motivation, lack of time, insufficient self-guidance, emotional avoidance, and difficulty understanding the assignments, as often noted in the literature as reasons for low adherence (Tang & Kreindler, 2017). Furthermore, a lack of parental support or concerns from therapists about the child’s ability to handle the homework, along with time constraints during the therapy session to discuss the homework (Kelly & Deane, 2011), may negatively affect homework practice in children. Therefore, it is important to consider these different factors when designing, delivering, and evaluating homework programs. Collaboration with children, parents, and therapists in the design of homework programs is essential. Additionally, providing therapists with effective training on delivering optimal treatment is important for the successful implementation of homework.

Finally, several methodological issues were mentioned that are informative to take into account when evaluating homework programs in future studies. There is, for example, some indication that homework in a specific stage of the treatment is more important than in other stages (Simpson et al., 2011; Westra & Dozois, 2007). Also, some studies found that focusing on qualitative aspects (such as quality of the homework) rather than on quantitative aspects (such as number of minutes spent on the homework) better predicted treatment outcome. Moreover, how homework adherence is measured (e.g., during treatment vs following treatment) may give different effects, and it seems a challenge to reliably measure homework adherence, especially in children. In this respect using an Mhealth technology, such as an app, could solve part of the problems as it automatically tracks the number of completed exercises, when the child spends time on homework and total time spent per activity.

To further facilitate the re-evaluation of current homework programs, eleven key elements, based on the most recent theoretical and empirical insights and research, were provided that are important to take into account when redesigning and evaluating homework for CBT programs for childhood anxiety disorders. As a first attempt to develop a homework program that includes all mentioned important elements, we developed the KibA homework program including an app for children with a specific phobia that could serve as an example of how homework could be improved. While developing this program, we learned three lessons that are important to take into account when developing a new homework program. First, the feedback from the child panel and therapist panel led to several major improvements in the program. As a result, we added more game elements, changed the layout to include more attractive colors, included more child-friendly instructions, and made the app more standalone with a monitoring role for therapists. Second, providing mock-up versions of the program in an early stage and the beta-versions quite early in the process helped the panels to visualize what the program could look like. This helped the panels to provide detailed feedback and recommendations for newer versions which was very helpful for the IT team that did not have any experience in making software especially for children with an anxiety disorder. Third, the close collaboration between researchers, IT specialists, and the end-users was instrumental in our end product. Researchers were crucial in order to provide theory-based features. IT specialists made sure the app was well designed and programmed and followed all privacy and data management requirements. The end-user feedback was crucial for user-friendliness. This qualitative feedback from all parties enhanced our understanding of the importance of co-developing a mHealth with end-users, and our understanding of the importance of multiple testing rounds to develop user-friendly mHealth apps (Barnum, 2020).

## Conclusion

The current review contributes to a growing body of support, suggesting the need for a critical re-evaluation of CBT programs for childhood anxiety disorders to enhance treatment outcomes (e.g., Craske et al., 2022; James et al., 2013; Levy et al., 2022). This review especially focused on the influence of homework on treatment outcome, recognizing that improving the quality of homework might have the potential to help children overcome their fears, reduce anxieties, and minimize relapse following treatment (Kazantzis et al., 2016). The lack of impact observed in current homework programs on treatment outcomes was explored, along with recommendations for future studies. To further guide the re-evaluation of current homework programs several recommendations for future studies were provided, along with a list of eleven key elements. These key elements, grounded in the most recent theoretical and empirical literature, are important considerations when (re-)designing and evaluating homework for CBT programs for childhood anxiety disorders.

As an example how to enhance the effectiveness of homework programs, we developed the KibA homework program, including an app, as part of the One-Session Treatment program commonly used to treat childhood-specific phobias. While we designed the app for children with a specific phobia, our app could easily be modified to incorporate homework for other anxiety disorders or other mental health disorders if found effective in our randomized clinical trial (Klein et al., 2023). Given that exposure is used as a treatment for various disorders, such as obsessive–compulsive disorder, panic disorder, separation anxiety, social anxiety, and more, the app might also facilitate home practice in these mental health disorders. Furthermore, with adjustments in program layout and instructions, the app might extend its utility to other age groups. The KibA homework program, including the app, is relatively standalone, with therapists adding individualized exercises and rewards via the online dashboard. This balance between autonomy and therapist involvement aims to support and motivate children while holding them accountable. In terms of future research, we hope that this review sets the stage and stimulates further investigation into redesigning and evaluating the effect of homework programs, including mHealth apps, in the treatment of various clinical child disorders.

## Figures and Tables

**Fig. 1:**
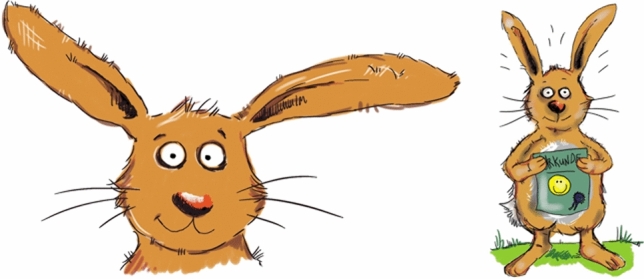
KibA mascot

**Fig. 2:**
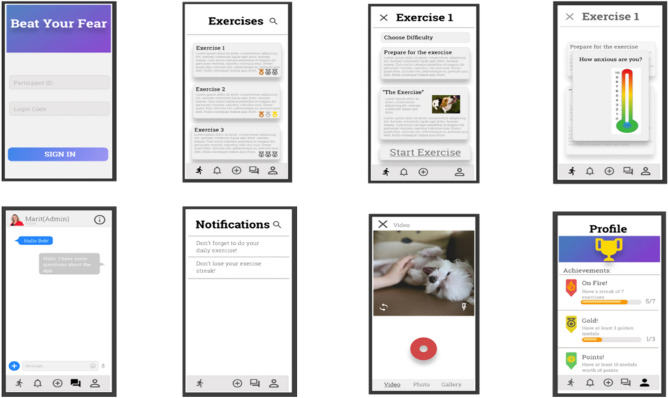
First mock-up

## Appendix A

### Theoretical factors

1. ***Setting the stage for expectancy violation*****.** To set the stage for expectancy violation in the KibA homework program, ten individualized exposure exercises with three difficulty levels are collaboratively developed by the therapist, the child, and with help of the parents during the homework preparation session.
  During the cognitive behavioral analysis, therapists explore the child’s dysfunctional beliefs regarding the phobic situation, identifying safety behaviors, and fear-enhancing factors. For instance, if a child fears dogs, the therapist delves into specific feared potential outcomes, coping mechanisms, and contextual nuances. This exploration aims to uncover the core fears in a detailed way. During the analysis, the therapist and child might progress from a general fear of dogs attacking to a more detailed fear scenario involving specific types of dogs, their actions, potential injuries, and environmental conditions (e.g., afraid that the dog notices me, runs at me, jumps and bites me in the face, leaving me in a lot of pain and needing a horrible surgery—This would most likely happen with bigger, active dogs that are off-leash with no other people between me and the dog).
  The subsequent in-session and home-practice exposure exercises strategically confront these fears, prompting expectancy violation by exposing the child to the feared stimulus (keeping in mind all the details from the analysis) without safety behaviors, so they can realize the feared outcome does not happen, and they can handle the situation. This process helps disconfirm dysfunctional beliefs, fostering the formation of new, non-catastrophic associations in memory (Craske et al., 2014). Each individualized exercise in the KibA homework app includes questions about anxious expectancies before and after the exercise.
2. ***Multiple contexts*****.** The fact that the therapist and the child together create the exercises helps to tailor the exercise for each specific situation. Exercises are created specifically to be performed in the natural daily environment of the child and are created in a way that they include multiple contexts. For example, a child with a dog phobia might do exercises that have to be performed in the street, a nearby park, an off-leash area for dogs, or at the house of a friend who has a dog. Additionally, more variation is added since each exercise has three difficulty levels. The difficulty levels often include small variations of the same exercise, for example, the same exercise with different people/alone, different durations, or at different times of the day. The set-up of the KibA homework program therefore facilitates practice in multiple contexts.
3. ***Flooding versus gradual exposure.*** As described in the main manuscripts in the key element section, it is not yet completely clear from a theoretical perspective if flooding or gradual exposure is most effective in working with children (de Jong et al., 2023). From a practical perspective, one of the most important aspects of homework is that children are able and willing to do the exercises at home, and practice often. In the KibA homework program, we have tried to combine the best of both worlds by including the 10 exercises in increasing level of difficulty, thereby facilitating a gradual progression through the exercises. However, the first three exercises (including the three difficulty levels per exercise) in the KibA app are available to the child when starting the program. The child is free to do these exercises in any order they want. If five out of the nine variations of the exercises are completed successfully, the next three exercises (with again its three difficulty levels per exercise) are unlocked. In this way, the progression through the exercises is somewhat gradual but also flexible, and children have a certain degree of autonomy to practice, but cannot just “skip” to the final most difficult exercise. Children are encouraged to do all exercises in the app at least once and are free to repeat them as many times as they want (and they are also reinforced to do so, by earning a ‘crown’ when completing all three difficulty levels of each exercise and ‘a shiny medal’ for each exercise they complete twice).
4. ***Mental rehearsal*****.** The KibA app includes a feature that is, to the best of our knowledge, unique among mental health care apps—it incorporates a photo and video option. During the four-week homework practice, children are encouraged to take pictures/videos of exposure experiences, storing these visuals to revisit. This process enhances their memory, with the expectation that it will increase feelings of self-efficacy (Beidas et al., 2010). In the KibA app, children can build a photo and video library of successful experiences, allowing them to review and share these moments with others. This functionality serves as a form of mental rehearsal, stimulating and reinforcing the retrievability of the newly formed non-catastrophic association, and it heightens motivation for future exposure exercises (McGlade & Craske, 2021).

### Methodological factors

5. ***Personalized exposure exercises*****.** The KibA homework program includes individualized exercises created by children and their therapists (with help from the parents). In this way, the program meets the different needs of the children and considers differences in experience, cognitions, and treatment goals. The homework program is designed to be used after an extensive case conceptualization is made and when the therapist and child have a good understanding of what situations are difficult for a child and what their feared expectations are.
6. ***Psychoeducation*****.** The KibA app includes short basic psychoeducation texts to provide easy access to information and topics that the therapist covered during treatment to support children with home practice. The app includes both written text and links to videos in which a therapist explains important psychoeducation topics for home practice, such as what to do when you are stuck on an exercise (step-by-step guide on how to get going again) and on what you can do if you feel really afraid after completing an exercise. Therapists also use psychoeducation when explaining the treatment rationale to children at the end of the cognitive analysis. During the massed exposure session, psychoeducation is often incorporated into the exposure exercises (e.g., during an exposure exercise with a dog, the therapist might point out the dog’s body language and help the child understand it and its behavior).
7. ***Contingency management*****.** The KibA app incorporates various features related to contingency management, integrating different game elements to increase app usage. These elements are based on operant conditioning principles, asserting that reinforced behavior is more likely to be repeated. Leveraging modern technology, such as gamification, has been shown to improve motivation and adherence to interventions (Dennis & O’Toole, 2014; Ferreira et al., 2014).
  For instance, In the KibA app, children can unlock sets of more challenging exercises by completing a specific number of the already accessible easier exercises, similar to the level design of mobile games. The difficulty levels of each exercise are represented by bronze, silver, or gold medals, which can be earned by completing the level, allowing for a gradual progression while offering flexibility for children to choose exercises and difficulty levels. A child can earn a crown when completing all three levels of an exercise. Digital coins are earned for successfully finishing exercises, achieving specific goals, such as taking pictures or videos of feared stimuli or situations and maintaining a streak of practicing for five consecutive days. The digital coins act as tokens in a ‘reward store,’ where children can purchase individualized rewards decided with their parents before the homework period (entered into the app by the therapist). Rewards may include small gifts or enjoyable activities to celebrate progress, such as extra screen time, a visit to the petting zoo, choosing a dessert, an additional bed-time story, or a festive picnic. Moreover, the KibA app features a dedicated progress page where children can view their earned medals and achievements, tracking their overall progress. The KibA app ‘game’ is completed by earning 25 out of the 30 medals. Upon program completion, the child receives a special surprise award predetermined by the parents in consultation with the therapist (e.g., dining out at the child’s favorite restaurant, a new video game, or a trip to the swimming pool). A child can earn a superhero card when doing an exercise again after not being able to complete one before. Finally, the app provides positive feedback through visuals, personal messages, and introduces a ‘mascot,’ the KibA-hare (see Fig. 1), designed to support the child throughout the program and enhance engagement.

### Child, Parent, and Therapist Factors

8. ***Therapist guidance and clear instructions*****.** The KibA homework program comes with a few features to ensure guidance and clear instructions. The app sends a daily reminder to practice at a time previously chosen by the child or parent. It also includes an online therapist dashboard through which the therapist can create an account for the child, include the exercises and rewards (which can be adapted during the homework period), and monitor the child’s progress. The therapist can use this information to discuss any difficulties during weekly scheduled phone calls with the child. Finally, the KibA app comes with clear instruction videos on how to use the app, has a ‘frequently-asked-questions’ section, and the exercises are made together with the child to ensure a good understanding of the exercises.
9. ***Self-monitoring*****. **All exercises in the KibA app come with a few questions before the start and after completion of each exercise. These questions address the level of fear and fear expectations, and how proud the children are of themselves for trying a certain exercise. These self-monitoring assessments help challenge cognitions and disconfirm dysfunctional beliefs. The assessments also allow children to see their progress throughout practicing and are helpful for the therapist to monitor the child’s progress.
10. ***Involvement of parents*****. **The One-Session Treatment and KibA homework program are child-centered interventions. However, parents are asked to play a supporting and facilitating role during the therapy and home practice period. The role of the parents during the homework period is discussed with the therapist, parents, and child prior to the homework period. Responsibility for completing the program lies with the child, but parents are encouraged to praise the child for steps that are made. Also, depending on the age, abilities, and wishes of the child, the parents may remind the child to practice, drive the child to practice locations, or arrange needed materials or situations as needed. If deemed appropriate by the therapist and in discussion with the child, parents may be asked to provide help with the exercises (at first). It is always stressed (especially with older children and adolescents) that the parents are not responsible for whether the child practices at home, but that the therapist will check on the progress of the child during the weekly phone calls.
11. ***Co-creation and user-friendliness.*** Recently, children have been assigned to a collaborative rather than consultative role in mHealth development (Jones et al., 2020). This shift states the importance of attention for and value of involvement of children as end-users in mHealth development. Our approach allowed different groups to test the app and IT specialists to adjust our app multiple times to ensure its usability. Children are not just little adults, and recognizing this distinction, we have designed our homework program to be more than a modified app originally created for adults. Our approach involved a user-centered iterative process, where children actively participated as co-developers. This methodology aligns with the guidelines emphasized by Jones et al. (2020). The KibA homework program including the app was created in co-creation and followed an extensive iterative process, including scientists, therapists, children, parents, and IT specialists (see also process of the development below). The design of the KibA homework program encourages shared decision-making when creating exercises with the child, includes clear instructions and specific exercises, has a user-friendly design, and the weekly phone calls, in which questions, possible dysfunctional attitudes and non-compliance can be addressed, facilitating the process. Also, the process of individualizing the content of the KibA homework program is conducted in close collaboration with each child and their parents during the treatment process.

## A comprehensive overview of the development of the KibA homework program

### Process of the Development of the KibA Homework Program

In the development of the KibA homework program, there was a significant emphasis on incorporating evidence-based features through a user-centered iterative design, aligning with key element 11 as described above. Feasibility and usability were tested multiple times, making a unique contribution to the mHealth field focused on childhood anxiety. Throughout the process, scientists, IT specialists, children, their parents, and therapists were actively involved.

After two meetings between the research team and the IT team, the first mock-up version of the app was available after a six-week building period by the IT team (See Fig. 2). An iterative process followed in which a panel of children (and their parents) reviewed the mock-up versions in five two-hour feedback sessions spread over nine months. Additionally, separate sessions with groups of different therapists were organized at different stages during this process. The primary goal of these sessions was to ensure the feasibility of the app. To achieve this, children, their parents, and therapist were included as end-users to ensure that they liked the app, wanted to use the app, and found the app useful. After each session, the IT team incorporated the feedback of the panels. In preparation for the first session, the child and parent panel had to think about apps they liked and to further brainstorm about it during the session. After brainstorming, session two was dedicated to giving feedback about the design (e.g., what should the app look like, which colors should be used). Session three centered on the reward system (e.g., what the users wanted to earn with exercises, how many points should be traded for a reward, and the reward store). During session four, the child panel tested the improved app in test-flight mode. After this session, the child panel tested the app at home and provided feedback in a telephone interview. The last session was the project evaluation (please see below for a detailed overview of each session). Figure 3 illustrates the finalized app.

After the first beta version of the app was created in collaboration with the child and therapist panels, a group of 14 children tested the app with pre-programmed generic exercises in a school setting. Their feedback was incorporated in a second beta version of the app, which was then tested with 8 children for a week at home. For this round of testing, personalized exercises for subclinical fears were used. Once again, feedback from the children and their parents was incorporated, leading to the development of a third beta version of the app, which underwent testing by two patients with a specific phobia, along with their parents and therapist. Feedback from all parties involved was incorporated into the final version of the KibA app, available in both the Google Play Store and the Apple Store as a therapist supported app. To assess the efficacy of the KibA homework program, including the app, the program is currently undergoing testing in a randomized controlled trial (see Klein et al., 2023).

## The Role and Goals of the Therapist and Child Panel Sessions

### Therapist Panels

At different stages during the development process, the mock-ups and beta-versions of the app were presented to groups of therapists from different clinics. These sessions were mostly set-up as open brainstorm sessions. Feedback from these sessions was used to further guide the development process.

### Overview Child Panel Sessions

The child panel consisted of 8 children and their parents***.*** The panel provided feedback on mock-ups in five 2-h sessions. Due to COVID-19, the sessions were not with the complete panel at once but in small subgroups.

#### Session 1 (Functionalities)

During the first session, children indicated that they wanted the app to resemble a game, and they wanted to achieve goals (like achievements in Mario Odyssey). In the final version of the KibA homework app this became pre-set goals, like “collect three medals” or “take five pictures” (Fig. 3). Furthermore, children reported they wanted a buddy system like the owl in Duolingo to feel supported while doing the exercises. In our app this became the KibA-hare that gives instructions, rewards at the end of an exercise, and encourages the child if an exercise is not fully completed (Fig. 3.2). Children also reported they wanted different levels like different games/levels in Gruffalo. In our app, this became three difficulty levels of each exercise (easy, medium, hard; Fig. 3.1). Lastly, children reported that the notifications were not specific enough and that they did not want to be notified by their therapist through a chat function. Therapists agreed because they did not think it was feasible to have a direct child–therapist connection all the time. We made the app more like a standalone app with therapists monitoring the progress via the therapist online dashboard and weekly phone calls, and one daily pop-up reminder message.

#### Session 2 (Design)

During the second session, children reported that the menu was unclear because the reward system and points were combined in one tab. Therefore, the IT team split the reward system and points in two separate tabs (Fig. 3.5 and 3.6). Children also reported that they missed colors and graphics in the app. In the final version, we used a bright green color and child-friendly graphics like medals, crowns, certificates, and points to make the app more suited to the age of end-users (Fig. 3.5).

#### Session 3 (Reward System)

During the third session, children reported that they preferred personalized rewards instead of virtual rewards. In our app, we included a store that contains ten personalized rewards that can be bought with earned points (Fig. 3.6). Points are earned with successful practice and reaching pre-set goals (added functionality from session 1, Fig. 3.3). Additionally, we added streaks (“Five days of consecutively practice”) to earn points (Fig. 3.4). A progress bar shows how close children are to finishing the game and to receive a surprise reward from their parents (Fig. 3.5). During this session, one child asked what would happen if children were not able to finish an exercise (i.e., if a child indicates that they did not complete an exercise or still has high anxiety levels). For these situations, we added “hero cards.” Children earn hero cards regardless of the outcome of exercises, to prevent them avoiding practicing due to earlier incomplete exercises or high levels of anxiety after an exercise (Fig. 3.2). Finally, we added “shiny” medals that children could earn by repeating exercises. These medals do not count for the total of 30 medals they can earn by doing all exercises. Nevertheless, we want to encourage and reward children for their effort for their practicing.

#### Session 4 and 5 (Test-Flight Modus and Project Evaluation)

All children tested the app in Test-Flight modus. This test environment allowed the IT team to distribute a test version of the app without launching the app. All children provided their feedback for the last time. Finally, researchers called all children for an interview to evaluate the project.

### Beta Testers at School

*Fourteen c*hildren tested the app for one hour at school, using pre-programmed generic exercises (e.g., get the jar with the bug from the researcher and hold the bug for 5 s), and rated the app and its functionalities and gave written and verbal feedback. The children rated the usability highly positive, while stigma was rated relatively low. Based on the experience in the classroom and the feedback from the children some technical bugs were fixed (problems with logging in and the media functionality). The children also emphasized that the reward store and game elements were very important to them.

### Beta Testers at Home

Eight children tested the app for one week at home and rated the app and its functionalities and gave written and verbal feedback. A researcher made personal exercises and rewards with the children and their parents. Since these were all children without an anxiety disorder diagnosis, most exercises were aimed at subclinical fears of the children or on schoolwork they had difficulty with. The children rated the usability highly positive, while stigma was rated relatively low. Two children encountered technical issues and these issues were addressed in improving the next version of the app. Based on the feedback of the children, some improvements were made to the layout (colors, size, and placement of buttons). Also the in-app texts – especially questions before and after each exercise - were shortened since children found these too long and boring. Based on parent feedback, some clarifications were made in the reward system and some messages were added (e.g., ask your parent if this is a good time to buy reward X).

### Clinical Cases

Two therapists tested the KibA program with two patients with a specific phobia following a one-session treatment. Feedback from patients, parents, and therapists was incorporated to create the current version of the KibA program. Most notably, some suggestions for therapists were added in the treatment manual (e.g., practical tips of already having a list of suitable exposure exercises that the child can choose and adapt from; using post-it’s to create the first version of the exercises so they can still be easily changed and swapped around before entering them into the app).
